# Epstein–Barr virus based biomarkers and targeted therapies in nasopharyngeal carcinoma: a narrative review

**DOI:** 10.1016/j.lanwpc.2026.101852

**Published:** 2026-08-17

**Authors:** Sen-Yu Feng, Ren-Jia Shu, Na Liu, Jun Ma

**Affiliations:** a Department of Radiation Oncology, State Key Laboratory of Oncology in South China, Guangdong Key Laboratory of Nasopharyngeal Carcinoma Diagnosis and Therapy, Sun Yat-sen University Cancer Center, Guangzhou 510060, PR China; b Department of Experimental Research, State Key Laboratory of Oncology in South China, Guangdong Key Laboratory of Nasopharyngeal Carcinoma Diagnosis and Therapy, Sun Yat-sen University Cancer Center, Guangzhou 510060, PR China

**Keywords:** Nasopharyngeal carcinoma (NPC), Epstein–Barr virus (EBV), EBV-related NPC biomarkers, EBV-targeted NPC therapies

## Abstract

Nasopharyngeal carcinoma (NPC) is a human malignancy with well-established association with Epstein–Barr virus (EBV) infection. Due to the concealed anatomical location of nasopharynx and the absence of early-stage clinical symptoms, most patients are diagnosed at a relatively advanced stage. EBV-related biomarkers, such as plasma EBV DNA and serum EBV antibodies, demonstrate outstanding sensitivity and specificity in the early detection of NPC. Moreover, plasma EBV DNA also serves as a useful tool for prognostic prediction of patients with NPC. Although current standard therapies have significantly improved the survival of patients with locoregionally advanced NPC, the establishment of personalized treatment plans remains challenging, and a subset of patients still experience disease progression even after definitive treatment. Plasma EBV DNA has shown great potential in guiding adaptive treatment of NPC, enabling optimum efficacy while minimizing treatment associated adverse effects. Additionally, EBV-targeted therapies, including cell therapies and small-molecule drugs activating anti-virus immune response or directly targeting EBV life cycle, represent promising approaches for these patients. In this review, we systematically summarized recent advances in EBV-targeted screening, prognostic prediction, adaptive treatment, and novel treatment strategies for NPC, discussed the strengths and limitations of these approaches and outlined future research directions in these fields.

## Introduction

Nasopharyngeal carcinoma (NPC) is a malignant tumor originating from the mucosal epithelium of the nasopharynx and exhibits distinct geographic prevalence. It is highly endemic in southern China and southeast Asia, with incidence in these endemic regions being 50 to 100 times greater than other parts of the world.^1^^,^^2^ The initiation and development of NPC are linked to carcinogenic environmental factors, genetic predisposition, and Epstein–Barr virus (EBV) infection.^3^ Notably, the incidence of developing NPC is significantly higher in individuals with a detectable plasma EBV DNA and EBV infection is almost exclusively observed in non-keratinizing NPC, which consists over 95% of cases in endemic regions.^2^^,^^4^^,^^5^ In non-endemic regions, however, keratinizing NPC constitutes over 75% of NPC and has a worse prognosis compared with non-keratinizing subtype.^6^^,^^7^ Besides, the overall EBV infection rate in these regions is significantly lower than endemic regions, while the EBV infection rate of Asian patients in these regions are still significantly higher than African American and White patients.^6^ A critical step in EBV-associated NPC carcinogenesis is the establishment of type II latency infection, during which EBV expresses virulence factors such as EBV-encoded small RNAs (EBER), EBV nuclear antigen 1 (EBNA1), latent membrane proteins (LMPs, including LMP1 and LMP2), and BamHI-A region rightward transcript miRNAs (miR-BARTs) and is capable of suppressing host immunity, promoting tumor cell survival, inducing genomic instability, reprogramming host cell metabolism, and driving de-differentiation of epithelial cells.^3^^,^^8^

In the clinical practice of NPC, three major challenges remain to be fully addressed: 1) how to identify high-risk populations and patients with early-stage NPC with higher sensitivity and accuracy; 2) how to evaluate clinical prognosis and dynamically conduct risk-adapted treatment strategies in patients with NPC; 3) how to improve the survival of recurrent and/or distant metastasis NPC as well as high-risk locoregionally advanced NPC who fails to benefit from existing combination therapy. Given the important role of EBV in the carcinogenesis of NPC and its high prevalence among patients with NPC in endemic regions, there is growing emphasis on the clinical applications of EBV-associated biomarkers and novel EBV-targeted therapy in solving the above-mentioned challenges, which have yielded encouraging results in screening, prognostic assessment, adaptive treatment, and rescue therapy of patients with NPC in both preclinical and clinical settings.

With a focus on Asia while also including research from other non-endemic areas, we will discuss recent progress in the application of EBV-related biomarkers, as well as summarizing advances in EBV-targeted NPC therapies in this review. We will also discuss current challenges and unresolved issues in these areas, along with perspectives on future research directions.

**Search strategy and selection criteria**

References related to EBV-related biomarkers and EBV-targeted therapies of NPC were comprehensively reviewed on PubMed, Web of Science, and Scopus, with search strategy including numerous keywords and their synonyms including Epstein–Barr Virus, EBV, nasopharyngeal carcinoma, NPC, screening, screening strategy, biomarkers, EBV DNA, EBV-DNA, EBV antibodies, prognosis, prognostic, prognostic prediction, prognostic assessment, adaptive treatment, dynamic treatment, personalized treatment, EBV-targeted therapy, cell therapy, autologous cell therapy, EBV-specific cytotoxic T lymphocytes, EBV CTLs, EBV-CTLs, tumor-infiltrating lymphocytes, tumor-infiltrating lymphocytes (TILs), chimeric antigen receptor T, CAR-T, T cell receptor-engineered T, TCR-T, small-molecule therapy, NPC vaccines, NPC vaccination, viral life cycle, anti-EBV immune response. Only papers published in English were reviewed.

## NPC Screening Strategies Based on EBV-related Biomarkers

The active EBV replication occurred 3–10 years before the clinical onset of NPC.^9^ The rationale for using EBV-related biomarkers, including serum EBV antibodies and plasma EBV DNA, in NPC detection lies in the distinct quantitative profiles or response patterns between individuals with NPC and those without. Plasma EBV DNA, which primarily reflects the activity of EBV replication and viral load, has outstanding efficacy in the detection of NPC, while serum EBV antibodies, which primarily reflecting the host's immune response status, offers a more practical and cost-effective approach for large-scale, standardized screening project.^10^, ^11^, ^12^, ^13^, ^14^, ^15^, ^16^ NPC screening strategies using single EBV-related biomarker or combining multiple EBV-related metrics with multistep testing continue to emerge and have demonstrated outstanding sensitivity and specificity^5^^,^^17^, ^18^, ^19^, ^20^, ^21^, ^22^, ^23^, ^24^ (Table 1, NPC Screening Strategies Based on EBV-related Biomarkers).

**Table 1:** NPC screening strategies based on EBV-related biomarkers.

Type	Time	Region	Number of participant	Age (year)	Sex (%)		Detection target	Early diagnosis rate (%)	Specificity (%)	Sensitivity (%)	Ref
					Male	Female					
Serum EBV Antibodies	2012	Sihui, China; Zhongshan, China	28,688	30–59	42·4	57·6	VCA-IgA + EBNA1-IgA	68·3	91·6	92·8	^17^
	2019	Sihui, China; Zhongshan, China	29,413	39–52	50·7	49·3	VCA-IgA + EBNA1-IgA	79·0	96·2	90·3	^18^
	2022	Sihui, China; Zhongshan, China	18,237	30–70	43·4	56·6	VCA-IgA + EBNA1-IgA	75·0	97·6	72·4	^19^
	2023	Zhongshan, China	24,852	30–69	40·3	59·7	P85-Ab	80·0	98·3	97·9	^20^
							VCA-IgA + EBNA1-IgA	79·0	97·0	72·3	
							P85-Ab + VCA-IgA + EBNA1-IgA	79·0	99·8	70·2	
Plasma EBV DNA	2017	Hong Kong, China	20,174	40–62	100·0	0·0	EBV DNA quantification	71·0	98·6	97·1	^5^
	2018	Hong Kong, China	20,174	40–62	100·0	0·0	EBV DNA quantification	–	97·4	97·1	^22^
							EBV DNA size	–	98·3	97·1	
							EBV DNA quantification + size	–	99·3	97·1	
	2019	Hong Kong, China	207	43–62	100·0	0·0	EBV DNA methylation	–	83·3	100·0	^21^
							EBV DNA quantification	–	75·0	100·0	
							EBV DNA size	–	35·8	100·0	
							EBV DNA methylation + quantification + size	–	94·2	100·0	

### EBV antibodies

Viral capsid antigen (VCA)-IgA is a serological marker indicative of the early stage of primary EBV infection, while EBNA1-IgA is detectable during the latent phase of EBV infection. By integrating the quantification of serum VCA-IgA and EBNA1-IgA levels, a risk-stratified screening program conducted in Sihui and Zhongshan, China, divided 28,688 Cantonese individuals into high-, medium-, and low-risk groups of developing NPC, with high-risk individuals referred to fiber-optic endoscopy assessment for confirmatory test.^17^^,^^18^ A subsequent clinical trial conducted in the same region introduced the quantification of nasopharyngeal EBV DNA load, obtained via nasopharyngeal swabs, into the risk classification system to determine whether a high-risk individual should be referred to endoscopy/MRI. After subdivision, only 63·4% of high-risk individuals were referred to confirmatory test, while the original specificity and sensitivity were preseved.^19^ After a 12 years follow-up, the screened cohort exhibited a 30% reduction in NPC-related mortality compared with the unscreened group.^25^

*BNLF2b* is an important lytic gene of EBV, while its biological functions remain largely unknown.^26^ The promoter of this viral protein, ED-L2, exhibits tissue-specific activity in squamous epithelia, which attributes to the tissue specific expression of this protein.^27^ Recently, a project conducted in Zhongshan, China, screened 24,852 individuals using serum antibodies targeting BNLF2b, including anti-P85-IgA, P85-IgG, and P85 total antibody (P85-Ab) identified 47 cases of patients with NPC, 38 of them were at an early stage, which validated P85-Ab as a promising biomarker for NPC screening.^20^ P85-Ab appears to be selectively elevated in NPC, as it was not detected in individuals with infectious mononucleosis or EBV-associated lymphomas in this study, indicating its disease specificity. Moreover, P85-Ab can identify potential NPC cases missed by the VCA/EBNA1-IgA assay. Its sensitivity of detecting early-stage disease is significantly higher than EBV DNA (96·4% vs. 78·6%), underscoring its substantial clinical value for the early detection of NPC. Compared with traditional serological biomarkers, the screening efficacy of P85-Ab alone is significantly higher than that of serum EBNA1-IgA, VCA-IgA, or dual-antibody combination approach (VCA-IgA + EBNA1-IgA), with higher sensitivity (P85-Ab 97·9% vs EBNA1-IgA 68·1%, VCA-IgA 55·3%, dual-antibody 72·3%), specificity (P85-Ab 98·3% vs EBNA1-IgA 95·1%, VCA-IgA 92·7%, dual-antibody 97·0%), and positive predictive value (PPV) (P85-Ab 10·0% vs EBNA1-IgA 2·6%, VCA-IgA 1·4%, dual-antibody 4·3%). Furthermore, the combination of P85-Ab with VCA/EBNA1-IgA further elevates the PPV to 44·6%. However, given the relatively short time of following up for only 6–18 months, the sensitivity and the specific screening strategies of P85 antibody, including the combination of other biomarkers, screening intervals, and follow-up schedules should be further validated based on long-term follow-up results.

### EBV DNA

#### Detection methodologies for EBV DNA

Beyond serological EBV antibodies-based screening strategies, EBV DNA also demonstrates great value in the early detection of NPC.^28^^,^^29^ The primary method for the clinical detection of EBV DNA is polymerase chain reaction (PCR). As early as 1989, PCR was used to detect EBV DNA in blood and salivary gland biopsy tissue.^30^ The advent of real-time quantitative PCR (qPCR) enabled precise measurement of viral load, and has gradually become the most commonly used method in the clinical detection of EBV DNA.^31^ Numerous factors, including PCR target selection and sample selection, greatly influence the detection sensitivity and specificity of EBV DNA, as well as its performance in NPC screening.

The multiple-repeat gene BamHI-W offers higher sensitivity than the single-copy gene EBNA-1. However, variations in BamHI-W copy number in different EBV isolates can potentially compromise assay comparability and standardization between laboratories. Moreover, the conversion ratio of BamHI-W copies to international unit might not always be accurate in different samples.^32^^,^^33^ Different sample types also influence the performance of EBV DNA detection and NPC screening. Plasma is the most commonly used sample for the detection of EBV DNA. However, a study conducted in Guangzhou indicated that the sensitivity and diagnostic performance of EBV DNA in nasopharyngeal swab outperformed plasma EBV DNA in the early detection of NPC.^34^ The diagnostic value of EBV DNA in nasopharyngeal brush is also superior to that of whole blood (WB) EBV DNA quantification.^35^ As for WB and different components of blood samples, a study conducted in Hawaii indicated that the detection sensitivity between WB and peripheral blood mononuclear cells (PBMCs) EBV DNA did not differ significantly and were both higher than that of plasma EBV DNA. A significant correlation was also observed between WB and PBMCs EBV DNA quantification, while both were less correlated with plasma EBV DNA.^36^, ^37^, ^38^ However, the efficacy of EBV DNA quantification in whole blood versus other blood components for NPC diagnosis has been rarely investigated. Well-designed head-to-head comparative studies are therefore warranted.

#### Plasma EBV DNA in NPC screening

As one of the most commonly used biomarkers in NPC clinical practice, the quantification and molecular characteristics of plasma EBV DNA shows outstanding efficacy in the early detection of NPC. A two-timepoint EBV DNA screening project was conducted in Hong Kong, China. Among the 20,174 participants, 1112 of them had detectable plasma EBV DNA and dual-timepoint positive EBV DNA occurred in 309 individuals. Among these 309 patients, 300 of them underwent confirmatory test and 34 were diagnosed as NPC, with the proportion of early-stage patients (stage I/II) significantly higher than that of the historical cohort. This project achieved 97·1% sensitivity and 98·6% specificity in NPC diagnosis, yielding a 90% relative improvement in progression-free survival (PFS).^5^ In 2023, 17,838 participants of this project underwent a second round of two-step screening, with slightly higher overall dual-timepoint EBV DNA positive rate and slightly lower NPC diagnosis rate compared with first round of screening. Participants with transient or persistent EBV DNA in the first round of screening had significantly higher probability of being identified as NPC in the second round of screening than those without, with relative risk of 16·8 and 4·4 respectively.^39^ Other than quantification of plasma EBV DNA, numerous molecular characteristics of circulating EBV DNA, including methylation profiles and fragmentation patterns, have also demonstrated substantial potential in NPC screening.^21^, ^22^, ^23^ A comprehensive fragmentomic analysis encompassing EBV DNA quantification, fragment size distributions, end motifs, and fragmentation-based methylation signatures, was conducted on 558 participants from a Hong Kong screening cohort with positive plasma EBV DNA in the first round of screening. The results indicate that individuals exhibiting distinctive mononucleosomal size, end motif types, and aberrated methylation profile of plasma EBV DNA have higher risk of developing NPC.^24^

### Comparison of EBV-related biomarkers in NPC screening

Although serum EBV antibodies-based and plasma EBV DNA-based screening strategies are both highly sensitive in early diagnosis of NPC in endemic regions, it is important to evaluate their screening efficacy among different clinical settings. There has been only one head-to-head comparison study conducted in Taiwan, China, indicated that plasma EBV DNA testing exhibited higher sensitivity and specificity than those of serum EBV antibodies for NPC detection, while both methods showed comparable sensitivity for detecting early-stage NPC.^15^ However, plasma EBV DNA screening was found to be more expensive and more dependent on advanced laboratory infrastructures than antibody-based methods.^16^ Therefore, to maximize screening efficacy, particularly for the detection of late-stage NPC, plasma EBV DNA testing is the preferred strategy. As for rapid and standardized population screening of early-stage NPC, both methods show comparable efficacy. However, when health economic evaluation and the feasibility of assay standardization are taken into account, EBV antibody-based screening strategy might represent a more practical option. To better define the clinical utility of these biomarkers in different clinical settings, more large-scale, multicenter, head-to-head clinical trials should be conducted to directly compare the sensitivity and specificity of serum EBV antibody-based and plasma EBV DNA-based screening strategies in diverse real-world settings, accompanied by comprehensive health economic evaluations, which are crucial for optimizing resource allocation especially in resource-limited regions. Furthermore, to establish optimal screening strategies tailored to different ethnic groups in both endemic and non-endemic areas, participants with diverse demographic characteristics and ethnic backgrounds should all be included.

## Prognostic assessment based on plasma EBV DNA

Given its strong association with treatment efficacy and long-term survival of patients with NPC, plasma EBV DNA has been widely recognized as a useful, accurate, and highly accessible biomarker for prognostic assessment. High level of plasma EBV DNA before treatment, at mid-treatment, end of treatment, and after treatment are all associated with significantly inferior clinical outcomes.^40^ Besides, dynamic evaluation of plasma EBV DNA level also yields outstanding prognostic prediction efficacy of patients with NPC.^41^

### Single time-point evaluation

Numerous studies have illustrated that high level of baseline plasma EBV DNA is significantly associated with inferior PFS and overall survival (OS) of patients with NPC, most of which were conducted in southeast Asia.^42^, ^43^, ^44^, ^45^, ^46^, ^47^, ^48^, ^49^, ^50^, ^51^, ^52^, ^53^ A clinical study conducted in Guangzhou, China, involving 8093 patients with NPC from stage I to IVa, found that patients with pre-treatment plasma EBV DNA higher than 2000 copies/mL had inferior OS.^49^ However, another study conducted in Fuzhou, China, which recruited 534 patients with NPC from stage III to IVa, revealed that patients with a baseline plasma EBV DNA higher than 6710 copies/mL had significantly worse 3-year OS, PFS, and distant metastasis free survival (DMFS).^52^ Apart from these two clinical trials, the optimal cut-off value of baseline plasma EBV DNA in prognostic assessment of patients with NPC remains inconsistent across numerous studies (Table 2, Different Cut-off Value of Baseline Plasma EBV DNA Level in Prognostic Assessment), which may be attributed to different demographic characteristics and clinical status of patients, different detection platforms and methods of plasma EBV DNA, the variability in study design, including sample sizes, endpoint definitions, and the statistical methods used to determine the cut-off value. To address this issue, standardization of detection methods, platform, and criteria for plasma EBV DNA testing is of utmost priority. Besides, sample selection, endpoint definition, and choice of statistical methodology also warrant further optimization. What's more, a comprehensive meta-analysis integrating existing studies should also be conducted. Beyond endemic areas, a retrospective study conducted in Italy further demonstrated that negative pre-treatment plasma EBV DNA is associated with significantly longer PFS of patients with NPC in non-endemic area.^47^

**Table 2:** Different cut-off value of baseline plasma EBV DNA level in prognostic assessment.

Year	Region	Median age (year)	Patient number	Sex		Patient stage	Research type	EBV DNA cut-off value	Ref
				Male (%)	Female (%)				
2002	Hong Kong, China	46	170	84·1	15·9	I–IV	Prospective	4000 copies/mL	^43^
2006	Hong Kong, China	47	376	74·0	26·0	I–IV	Retrospective	4000 copies/mL	^44^
2007	Taiwan, China	46	152	70·4	29·6	I–IV	Retrospective	1500 copies/mL	^45^
2016	Guangzhou, China	44	584	74·7	25·3	I–IVb	Retrospective	2010 copies/mL	^46^
2017	Milan, Italy	48.5	130	66·9	33·1	II–IVb	Retrospective	3493 copies/mL	^47^
2017	Bangkok, Thailand	49	204	73·5	26·5	I–IVb	Retrospective	600 copies/mL	^48^
2020	Guangzhou, China	45	8093	70·3	29·7	I–IVa	Retrospective	2000 copies/mL	^49^
2020	Taiwan, China	–	52	84·6	15·4	IV	Retrospective	3500 copies/mL	^50^
2021	New Delhi, India	25	25	52·0	48·0	II–IVb	Retrospective	1500 copies/mL	^51^
2022	Fuzhou, China	48	534	77·0	23·0	III–IVa	Retrospective	6710 copies/mL	^52^
2022	Guangzhou, China	46	190	82·7	17·3	–	Prospective	10,000 IU/mL	^53^

Apart from baseline, plasma EBV DNA level at numerous other time-points also yield prognostic prediction efficacy. Positive plasma EBV DNA after induction chemotherapy is strongly associated with inferior clinical outcome of patients with NPC, while an early clearance of plasma EBV DNA after induction-phase was associated with prolonged survival.^54^, ^55^, ^56^, ^57^ During intensity-modulated radiation therapy, patients with half-life clearance of plasma EBV DNA >15 days had worse 3-year PFS, DMFS, and OS.^58^ Furthermore, plasma EBV DNA ≥500 copies/mL within 0–2 weeks or a detectable plasma EBV DNA four weeks after radiotherapy is associated with higher rate of distant metastasis and shorter PFS and OS of patients with NPC. Notably, a detectable plasma EBV DNA at 8–12 weeks after radiotherapy is an even stronger indicator of disease relapse.^59^, ^60^, ^61^

### Dynamic evaluation

Longitudinal monitoring of plasma EBV DNA at multiple time-points during therapy can also provide highly informative data on the prognosis of patients with NPC.^62^^,^^63^ A pivotal clinical trial involving 673 patients with NPC in Guangzhou demonstrated that on-treatment plasma EBV DNA kinetics can be categorized into eight distinct patterns, which can be further classified into four prognostic phenotypes, highlighting the superior prognostic value of real-time plasma EBV DNA monitoring over conventional single time-point measurement.^64^ This concept was subsequently validated and further refined in the large-scale prospective EP-SEASON study with more detailed characterization of plasma EBV DNA dynamics, which confirmed the previously defined prognostic phenotypes and further stratified “late responders” into distinct subgroups based on long-term survival, leading to the identification of five whole-course circulating tumor DNA changing dynamics (WCCD) patterns.^65^ Collectively, these studies underscore a paradigm shift from static prognostic assessment based on single time-point plasma EBV DNA quantification toward a dynamic, response-adapted evaluation framework, thereby paving the way for more precise and individualized prognostic stratification in the future.

### Multiparameter evaluation

Given the prognostic prediction value of plasma EBV DNA, multiple studies have combined it with tumor, nodes, metastasis (TNM) staging or image-based criteria to develop comprehensive prognostic evaluation systems.

The recursive partitioning analysis (RPA) model based on post-induction chemotherapy plasma EBV DNA with overall TNM stage can divide patients with NPC into three risk groups with distinct DMFS, OS, and PFS.^66^ Besides, combination of post-radiotherapy plasma EBV DNA level and TNM stage also yields prognostic classification efficacy.^67^ These classification models had better risk discrimination efficacy than either the TNM or plasma EBV DNA alone.

Apart from TNM staging, studies from Hong Kong and Guangzhou both found that integrating tumor radiographic parameters with plasma EBV DNA level also provides potent prognostic prediction efficacy. Patients achieved plasma EBV DNA clearance within ten days and more than 50% drop in sum of maximum standardized uptake value of dual fluorodeoxyglucose positron emission computed tomography four weeks after starting chemotherapy had better OS and PFS.^68^ More recently, based on different cut-off values of gross tumor volume and gross tumor volume of nodes post-induction chemotherapy and plasma EBV DNA level post-induction chemotherapy and post-CCRT, patients with NPC can be divided into three groups with distinct long-term survivals.^69^

However, current prognostic studies combining plasma EBV DNA with either TNM staging or radiographic parameters are mostly retrospective. The standardized workflow of plasma EBV DNA detection was yet to be established and there was also no consensus on the optimal plasma EBV DNA cut-off value or detection time. As for image-based evaluation, the assessment of primary tumor volume and regression status is particularly challenging and subjective due to the complex anatomical structure of nasopharynx and the lack of uniformed assessment workflow. More standardized or artificial intelligence-guided imaging evaluation methodologies are still needed. Besides, which radiographic parameters, when combined with plasma EBV DNA quantification, have the optimal prognostic prediction efficacy remains unclear and necessitates further comparative studies.

## Plasma EBV DNA-guided adaptive treatment

Adaptive treatment is de-escalation or escalation of standard treatment therapy based on the alterations of certain biomarkers, with the aim of achieving optimum therapeutic outcomes while maintaining patient quality of life and improving cost-effectiveness. Evolving from single-phase treatment adjustments based on single timepoint plasma EBV DNA level to real-time treatment modifications following dynamic monitoring of plasma EBV DNA, the efficacy of plasma EBV DNA-guided adaptive treatment has been greatly improved.

### Induction chemotherapy and CCRT

CCRT constitutes the backbone of treatment for patients with locoregionally advanced NPC, while the addition of induction chemotherapy further improves their failure-free survival (FFS) and OS.^70^ However, whether all these patients should undergo induction chemotherapy followed by same intensity of CCRT remains unknown. To address this question, several studies conducted in Guangzhou showed that plasma EBV DNA, which is strongly relevant to prognosis and disease progression of NPC, can act as an effective risk-stratification biomarker to address this issue.

For patients with pre-treatment plasma EBV DNA <4000 copies/mL, 5-year OS is comparable regardless of the addition of induction chemotherapy. Moreover, reduced-dose radiotherapy at 60 Gy achieves similar 2-year PFS compared with the standard 70 Gy regimen for these patients, with lower treatment-related toxicities.^71^^,^^72^ Besides, the efficacy of two-cycle 100 mg/m^2^ concurrent cisplatin (DDP) regimen is also non-inferior to three-cycle, but with significantly less grade 3–4 mucositis, hyponatremia, and dermatitis.^73^ If these patients achieve an undetectable plasma EBV DNA during the second cycle of DDP (mid-DNA), the efficacy of receiving ≤200 mg/m^2^ cumulative cisplatin dose (CCD) does not differ from those receiving >200 mg/m^2^ CCD, but with significantly fewer all-grade late toxicities. In contrast, >200 mg/m^2^ CCD is more suitable for high-risk patients with pre-treatment plasma EBV DNA ≥4000 copies/mL or having a detectable mid-DNA in order to achieve satisfactory survival outcomes.^74^ For patients with pre-treatment plasma EBV DNA ≥1500 copies/mL, intensification treatment involving neoadjuvant treatment with the programmed death receptor 1 (PD-1) inhibitor and CCRT can improve their PFS compare with CCRT monotherapy.^75^ Furthermore, other ongoing clinical trials are further exploring the potential of EBV DNA-guided adaptive regimens of induction therapy and CCRT (NCT04223024, NCT05628922, NCT05772208).

### Adjuvant chemotherapy

Adjuvant chemotherapy can significantly prolong the 5-year PFS of patients with detectable plasma EBV DNA in midpoint-radiotherapy, but not for patients with undetectable plasma EBV DNA.^76^ For patients with persistently detectable plasma EBV DNA during follow-up, the employment of adjuvant chemotherapy should be considered, especially for patients with pretreatment plasma EBV DNA load ≥4000 copies/mL or a detectable plasma EBV DNA ≥1200 copies/mL during follow-up.^77^ Specifically, adjuvant capecitabine (1000 mg/m^2^ twice a day for 14 days every three weeks for eight cycles) following CCRT can significantly prolong the FFS of patients with pre-treatment plasma EBV DNA titer higher than 20,000 copies/mL compared with CCRT alone group with tolerable adverse events.^78^ There is also a clinical trial investigating the efficacy of combining capecitabine with tislelizumab in treating patients with residual plasma EBV DNA after radiotherapy (NCT07067268).

### Dynamic evaluation

Beyond putting single timepoint plasma EBV DNA level into consideration, numerous adaptive treatment evaluation systems have been established, which implemented a stratified intervention strategy guided by the dynamic changes of plasma EBV DNA level. This strategy offers more comprehensive monitoring of treatment response and greater flexibility in treatment modification, which consequently enhances therapeutic efficacy while minimizing treatment-related adverse effects and reducing the financial burden of patients with NPC.

Based on the aforementioned four phenotypic model and WCCD patterns, on-treatment plasma EBV DNA dynamic based, risk-adapted, individualized treatment strategies were preliminarily constructed, which can significantly improve patient survival while minimizing treatment-related adverse effects.^64^^,^^65^ Based on this theory, a phase II, multicenter, plasma EBV DNA guided EP-STAR trial was designed. In this trial, patients who achieved sustained clearance of plasma EBV DNA after the first cycle of induction chemotherapy were classified as low-risk and should continue with standard CCRT. While for those with plasma EBV DNA clearance in the second or third cycle, or with detectable plasma EBV DNA after induction chemotherapy, a risk-based treatment adaptation, from the addition of adjuvant metronomic capecitabine to intensification with 12 cycles sintilimab during and after CCRT should be activated. This adaptive therapy significantly improved the 3-year FFS of locoregionally advanced NPC with manageable toxicity compared with a contemporaneous external cohort.^79^ Moreover, numerous randomized controlled trials investigating the optimal treatment plans for patients with NPC based on plasma EBV DNA dynamics are still underway (NCT02135042, NCT05517135).

## EBV-targeted therapies

**Fig. 1:**
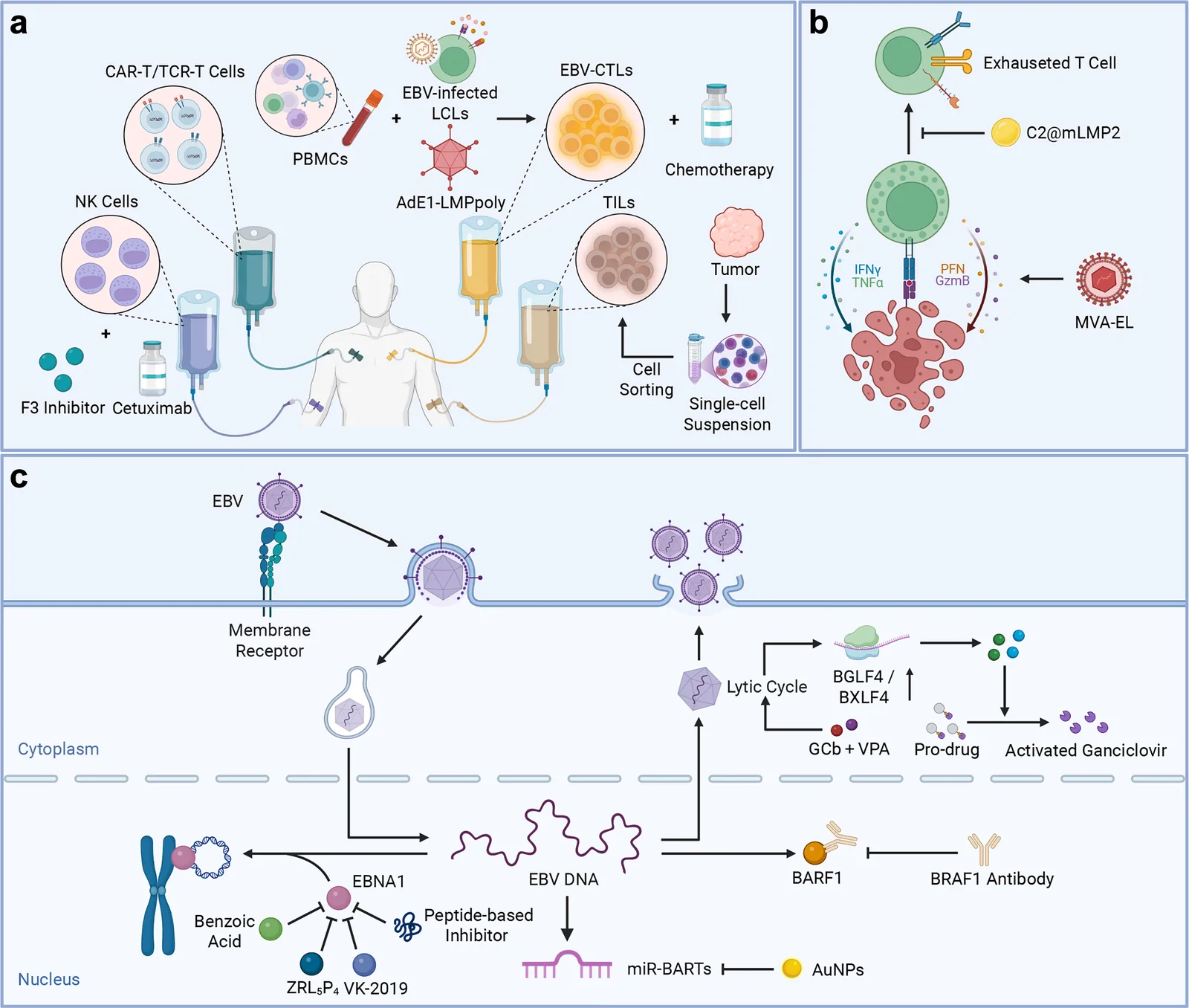
**Summary of EBV-targeted NPC Therapies.** EBV-targeted NPC therapies can be broadly categorized into three types. **a.** Cell therapies, including CAR-T/TCR-T and autologous cell therapies such as EBV CTLs, TILs, and NK cells, which can be used together with chemotherapy or immune therapy. **b.** The second type is designed to activate antiviral immune responses or reverse T-cell exhaustion, thereby enhancing the elimination of EBV-infected tumor cells. **c.** The third type involves agents that directly target viral proteins or mRNA to interrupt the EBV life cycle, thereby inhibiting viral replication and potentiate the elimination of EBV-positive tumor cells. F3, human tissue factor 3; NK Cells, natural killer cells; CAR-T cells, chimeric antigen receptor T cells; TCR-T cells, T cell receptor-engineered T cells; PBMCs, peripheral blood mononuclear cells; EBV, Epstein–Barr virus; LCLs, lymphoblastoid cell lines; AdE1-LMPpoly, adenoviral vector-based vaccine; EBV-CTLs, EBV-specific cytotoxic T lymphocytes; TILs, tumor-infiltrating lymphocytes; C2@mLMP2, LMP2-mRNA lipid nanoparticle; MVA-EL, modified vaccinia virus Ankara recombinant; EBNA1, EBV nuclear antigen 1; ZRL_5_P_4_, EBNA1 inhibitor containing an EBNA1 binding peptide with a Zn^2+^ chelator; VK-2019, a small molecule EBNA1 inhibitor; miR-BARTs, BamHI-A region rightward transcript miRNAs; AuNPs, gold nanoparticles; BARF1, BamHI-A rightward frame 1; GCb, gemcitabine; VPA, valproic acid.

More than two-thirds of patients with NPC were diagnosed with advanced-stage diseases.^80^ Currently, the standardized treatment for locoregionally advanced NPC is induction chemotherapy followed by CCRT.^70^^,^^81^, ^82^, ^83^ Combination of immune therapy further improves patient outcome.^84^^,^^85^ However, there is a subset of patients who still fail to benefit from existed combination therapy.^70^^,^^81^ Nowadays, with growing insight into virulence factors of EBV and the development of biopharmaceuticals, EBV-targeted NPC therapies, including cell therapies and small-molecule drugs activating anti-virus immune response or directly targeting EBV life cycle, have emerged as highly promising therapeutic strategies for patients with refractory or recurrent locoregionally advanced NPC (Fig. 1, Summary of EBV-targeted NPC Therapies).

### Cell therapy

In recent years, cell therapy has become an increasingly important modality in cancer treatment, particularly for hematologic malignancies.^86^^,^^87^ Despite its application in solid tumors remains in early phase, encouraging results have been achieved in some clinical trials, indicating its promising future.^86^ For NPC, most cell therapy products, including chimeric antigen receptor T (CAR-T), T cell receptor-engineered T (TCR-T), EBV-specific cytotoxic T lymphocytes (EBV-CTLs), and other cell therapy regimen, were designed to target numerous virulence proteins of EBV, with encouraging preliminary clinical results (Table 3, Summary of Clinical Trials of EBV-targeted NPC Therapies).

**Table 3:** Summary of clinical trials of EBV-targeted NPC therapies.

Year	Phase	Type	Region	Major antigen	Patient stage	Adverse effect	Patient number	Efficacy	Ref
2001	Phase Ⅰ	Autologous EBV CTLs	Hong Kong, China	Multiple EBV antigens	Metastatic or locoregional recurrence stage IV/V NPC	–	4	No assessment	^88^
2004	Case Report	Allogeneic EBV CTLs	Pavia, Italy	LMP2	Locoregional recurrence stage IIb NPC	Temporary cranial nerves II and IV impairment	1	Temporary stable, eventually progression	^89^
2005	Phase Ⅰ	Autologous EBV CTLs	Pavia, Italy	LMP2	Metastatic or stage IV NPC	2 patients with inflammatory reaction in the disease site	10	2 partial response (PR), 4 stable disease (SD), 4 progressive disease (PD)	^90^
2010	Phase Ⅰ/Ⅱ	Autologous EBV CTLs	Houston, USA	BZLF1, EBNA1, EBNA3s, LMP1, LMP2	Recurrent/refractory stage III/IV NPC	1 patient with bulky disease and pre-existed facial swelling	23	Remission patients: 5 remain remission, 3 recurrent disease; Active disease patients: 3 complete response (CR), 2 PR, 3 SD, 5 PD, 2 CR undetermined	^91^
2012	Phase Ⅰ	Autologous EBV CTLs	Hong Kong, China	LMP1, LMP2, EBNA1	Distant metastatic or locoregional recurrence NPC	Multiple grade 1/2 adverse effects, a single case of a serious adverse effect unrelated to treatment	24 enrolled, 14 treated	Median OS increased from 220 to 523 days	^92^
2014	Phase Ⅱ	Autologous EBV CTLs	Singapore	BZLF1, BRLF1, BRMF1, EBNA3s, LMP1, LMP2	Recurrent/refractory NPC	Multiple grade 1/2 adverse effects	38	3 CR, 22 PR, 10 SD	^93^
2017	Phase Ⅰ/Ⅱ	Autologous EBV CTLs	Boston, USA	EBNA1 and LMP2	Recurrent/PD after initial treatment or distant metastasis NPC	Multiple grade 1/2 adverse effects, 1 grade 3 adverse effect, 1 grade 5 adverse effects unrelated to EBV CTLs	21	1 CR, 2 SD, 18 PD	^94^
2021	Case Report	Autologous EBV CTLs	Queensland, Australia	FLYALALLL, CLGGLLTMV, LTAGFLIFL	Metastatic stage IV NPC	–	1	CR	^95^
2023	Retrospective study	Autologous EBV CTLs	Pavia, Italy	–	Recurrent/metastatic NPC	2 grade 1 flu-like syndrome	16	–	^96^
2024	Phase III	Autologous EBV CTLs	Singapore; Malaysia; Taiwan, China; Thailand; USA	–	Untreated metastatic/locally recurrent NPC	13 treatment-emergent adverse events (TEAE), 1 grade 2 serious adverse effect, 1 grade ≥ 3 TEAE	330	No significant prolonged OS of adding CTL to GC therapy	^97^
2015	Phase Ⅰ	Autologous TILs	Guangzhou, China	LMP1, LMP2, EBNA1	Stage III/IV NPC without distant metastasis	Grade 3 neutropenia	23 enrolled, 20 treated	1 relapse, 1 PD, 18 CR	^98^
2023	Phase Ⅱ	Autologous TIL	Guangzhou, China	–	Stage III/IV NPC without distant metastasis	4 grade 2 allergic reaction	156	No significant prolonged 3-year PFS rate of adding CTL to CCRT	^99^
2024	Phase Ⅰ	CAR-T	Guangzhou, China	Gp350	Advanced metastatic NPC	No severe adverse effects	11	Efficient disease control, tumor shrinkage and descend plasma EBV DNA	^100^
2013	Phase Ⅰ	MVA-EL	Hong Kong, China	EBNA1, LMP2	Stage I-IV NPC patients finished first-line treatment and were in remission	Multiple grade 1 adverse effects, 2 grade 2 adverse effects, 1 grade 3 adverse effects	18	No assessment	^101^
2012	Pilot Study	CLVA	Amsterdam, Netherlands	–	Residual, recurrent, and metastatic EBV-positive NPC	1 grade 4 and 1 grade 3 platelet count decrease, 1 grade 3 anorexia, aspiration pneumonia, infections and hepatotoxicity	3	Significant decrease of tumor in the nasopharynx	^102^

#### CAR-T and TCR-T

CAR-T cell therapy and TCR-T cell therapy can directly engineer CD8^+^ T cells to recognize and eliminate tumor cells expressing EBV-specific antigens. Preclinical studies have demonstrated that LMP1-specific CAR-T cells can effectively eliminate LMP1-expressing NPC cells and reduced tumor burden in mouse models.^103^^,^^104^ LMP1-specific TCR-T also inhibited LMP1-expressing tumor growth in mouse models, as did LMP2-specific TCR-T cells.^105^^,^^106^ EBV glycoprotein 350 (gp350) is a key surface protein that mediates the attachment of EBV to host cells.^107^ A phase I clinical trial recently conducted in Guangzhou using anti-gp350 CAR-T (BRG01) to treat advanced metastatic NPC demonstrated promising efficacy. BRG01 treatment is well tolerated and is capable of achieving tumor de-escalating and efficient disease control in patients with NPC, with significantly decreased plasma EBV DNA level.^100^ Numerous clinical trials discovering the treatment efficacy and safety backgrounds of EBV targeting CAR-T/TCR-T cell therapies with known targets are still underway (Table 4, Clinical Trials of NPC Cell Therapy).

**Table 4:** Clinical trials of NPC cell therapy.

Clinical trial ID	Region	Beginning time	Estimated finishing time	Patient number	Phase	Disease	Treatment	Targets	Target origin
NCT02980315	Nanjing, China	2016/11/01	2017/12/01	20	Phase Ⅰ/Ⅱ	EBV positive NPC	CAR-T	LMP1	EBV
NCT03925896	Guangzhou, China	2019/08/07	2022/08/01	27	Phase Ⅰ	Recurrent/metastatic EBV positive NPC	TCR-T	LMP2	EBV
NCT03648697	Fuzhou, China	2018/10/10	2021/10/10	20	Phase Ⅰ/Ⅱ	Refractory EBV positive NPC	TCR-T	LMP1/LMP2/EBNA1	EBV
NCT04509726	Chongqing, China	2023/03/01	2026/01/15	9	Phase Ⅰ/Ⅱ	Recurrent/metastatic EBV positive NPC	TCR-T	LMP2	EBV
NCT02065362	Houston, USA	2015/02/01	2033/02/01	14	Phase Ⅰ	Recurrent/refractory EBV positive NPC	Autologous EBV CTLs	LMPs/EBNA1/BARF1	EBV
NCT01447056	Houston, USA	2012/02/01	2020/01/22	37	Phase Ⅰ	Recurrent/refractory EBV related diseases	Allogeneic EBV CTLs	LMP1/LMP2	EBV
NCT02287311	Houston, USA	2015/02/01	2029/03/01	38	Phase Ⅰ	Recurrent/refractory EBV related diseases	Allogeneic EBV CTLs	LMPs/EBNA1/BARF1	EBV
NCT03044743	Nanjing, China	2017/04/07	2022/03/01	20	Phase Ⅰ/Ⅱ	Stage IV EBV positive gastric carcinoma, NPC, and lymphoma	Autologous EBV CTLs	–	–

#### EBV-CTLs

EBV-CTLs are autologous or allogeneic adoptive T-cells generated from peripheral blood of individuals without genetic modification. A classic approach of generating EBV-CTLs involves the direct stimulation of PBMCs with EBV-transformed lymphoblastoid B-cell lines (LCLs) that express high levels of EBV antigens, thereby activating and inducing the expansion of the EBV-specific T cells until a sufficient number of EBV-CTLs is obtained for infusion.^108^ A clinical trial reported control of disease progression was obtained in six out of ten patients with stage IV NPC receiving intravenous autologous EBV-CTLs infusion after conventional chemoradiotherapy.^90^ EBV-CTLs can induce long-term LMP2-specific immune responses, increase the number of tumor-infiltrating CD8^+^ T cells and safely reduce plasma EBV DNA to undetectable levels in patients with NPC.^88^^,^^89^ Besides, the proportion of LMP2 targeting T cells in EBV-CTLs product positively correlated with patient OS, underscoring the importance of LMP2-directed immunity in anti-tumor immune response of NPC.^93^ Patients undergoing EBV-CTLs immunotherapy targeting EBV nuclear antigens and LMPs also show a durable response.^94^ A meta-analysis further confirmed improved OS and a favorable safety profile across multiple EBV-CTLs trials of NPC.^109^ Apart from LCLs, stimulation of PBMCs with AdE1-LMPpoly, an adenoviral vaccine encoding multiple CD8^+^ T cell epitopes from EBNA1, LMP1, and LMP2, is another effective way of generating EBV-CTLs, which overcomes the antigenic bias of traditional LCLs stimulation method by targeting viral antigens truly expressed in tumor cells and enables more efficient expansion of EBV-CTLs.^110^^,^^111^ Clinical evaluation of this method in Hong Kong successfully expanded EBV-CTLs in 16 out of 24 patients with NPC.^92^ Infusion of AdE1-LMPpoly stimulated EBV-CTLs stabilized patients with relapsed and refractory NPC and these patients achieved a prolonged OS without significant toxicity, indicating its clinical utility.^92^^,^^112^

The efficacy of EBV-CTLs treatment is inversely associated with the level of myeloid-derived suppressor cells (MDSCs), while chemotherapy is capable of constraining the expansion of MDSCs, thus facilitating the tumor eliminating function of EBV-CTLs.^113^ A phase I clinical trial containing patients with recurrent or metastatic NPC received first-line chemotherapy followed by two doses of EBV-CTLs demonstrated promising efficacy.^96^ A phase II clinical trial showed that four cycles of gemcitabine and carboplatin followed by six doses of EBV-CTLs achieved a response rate of 71·4%, with 2-year and 3-year OS rate of 62·9% and 37·1%, respectively.^93^ Apart from traditional chemotherapy or radiotherapy, a case report demonstrated complete metastatic resolution and 22 months relapse-free survival in a patient receiving PD-1 blockade after infusion of EBV-CTLs, indicating the existence of potential synergistic effect.^95^

#### Other types of cell therapy

Apart from CTLs, expansion and infusion of TILs is another promising strategy of NPC cell therapy. In a phase I clinical trial conducted in Guangzhou, TILs infusion following CCRT showed promising anti-tumor efficacy, with 19 of 20 patients exhibited an objective antitumor response and an elimination of plasma EBV DNA.^98^ NK cell therapy also yields anti-tumor efficacy, yet with relatively less attention. An open-label phase I clinical trial conducted in Singapore demonstrated that infusion of in vitro expanded autologous NK cells following the administration of cetuximab led to the successful expansion of NK cell without intolerable adverse effects. Among seven stage IV patients with NPC who failed at least two lines of prior chemotherapy, stable disease was observed in four patients and progressive disease in three patients.^114^ Besides, a case report demonstrated that in a patient with advanced NPC and intracranial metastases who showed progression during conventional treatment, infusion of in vitro expanded allogeneic NK cells successfully decreased the intracranial metastases 31 months after treatment and the tumor response indicated partial response.^115^ Furthermore, recent studies found that EBV protein LMP2A upregulates human tissue factor (F3), thus impairing NK cell function. Combination of an F3 inhibitor with adoptive NK cell infusion showed significant efficacy in NPC mouse model.^116^ Compared with the above-mentioned cell therapies, the anti-tumor effect of autologous dendritic cells (DCs) transduced with Ad-ΔLMP1-LMP2 seems limited. Therefore, future studies should focus on enhancing its anti-tumor potency and developing DCs vaccine that targets patients with lower tumor burden.^117^

#### Conflicting clinical evidence behind cell therapy

Despite the aforementioned success of EBV-CTLs/TILs in treating patients with NPC, conflicting results still exist. A multicenter, randomized, phase III clinical trial conducted across thirty clinical sites in Singapore, Malaysia, Taiwan China, Thailand, and the USA containing 330 patients with recurrent or metastatic NPC with positive plasma EBV DNA indicated that the addition of EBV-CTLs infusion to gemcitabine and carboplatin has a favorable safety profile but could not improve the OS compared with chemotherapy alone.^97^ Besides, another clinical trial conducted in Guangzhou containing 167 patients with locoregionally advanced NPC with pretreatment plasma EBV DNA ≥4000 copies/mL also demonstrated that TILs infusion plus CCRT could not improve the 3-year PFS than CCRT monotherapy.^99^

There are several potential reasons for the unsatisfactory efficacy of these autologous cell therapies. Although well-tolerated, the overall adverse events associated with autologous cell therapies were still higher than those in the traditional chemotherapy/CCRT-alone group, which led to reduced patient tolerance and lower treatment completion rates, thereby compromising the efficacy of autologous cell therapies. Additionally, shorter cycle of combined chemotherapy in the autologous cell therapies group may have also contributed to the inferior outcomes. Another factor that probably devastates the efficacy of autologous cell therapies is their limited intratumoral infiltration. Therefore, more prospective, well designed, two-arm randomized controlled trials using low-toxicity, high efficacy autologous cell therapy products are still needed to clarify their efficacy and the synergistic effect of combination with traditional chemotherapy or radiotherapy (Table 4, Clinical trials of NPC cell therapy).

### Other EBV-targeted therapies

With deeper insights into virulence factors and life cycle of EBV, an increasing number of small molecules have been identified that can either directly disrupt key steps of viral life cycle or activate anti-EBV immune responses, thus interrupting the infection of EBV and leading to the elimination of EBV-positive host cells (Table 3, Summary of Clinical Trials of EBV-targeted NPC Therapies).

Directly targeting viral proteins or mRNA essential for EBV life cycle or inducing EBV lytic infection with small molecules is a promising strategy, although current studies remain largely at the preclinical stage. EBNA1 inhibitor, including benzoic acid and peptide-based inhibitor, can disrupt the DNA binding efficacy of EBNA and interfere with its homodimerization, inhibiting EBV latent infection and suppressing the growth of EBV^+^ tumor in preclinical NPC model.^118^, ^119^, ^120^, ^121^, ^122^ An EBNA1 inhibitor (ZRL_5_P_4_), containing an EBNA1 binding peptide with a Zn^2+^ chelator can enter EBV^+^ tumor nuclei, disrupt EBNA1 oligomerization and transactivation, reduce viability of NPC cells, and delay the growth of NPC xenograft.^123^ Recently, a clinical trial involving 23 metastatic/recurrent patients with NPC validated the safety profile of an EBNA1 inhibitor, VK-2019. Although only one patient achieved partial response, the biological activity of VK-2019 was evident. Eight patients exhibited a decline of post treatment plasma EBV DNA levels. Besides, analysis of tumor biopsies revealed a significant reduction in EBV genomic copy numbers within tumor cells, along with decreased expression of viral genes such as EBER. Furthermore, downregulation of host genes including CXCL10 and CD74, and upregulation of CXCL8 (IL-8) were observed, confirming the on-target biological efficacy of this small molecule inhibitor.^124^ Future optimization of the dosing regimen may be required to enhance its antitumor effects. miR-BARTs contribute to the latency infection stage, immune escape, and tumor proliferation of EBV.^125^, ^126^, ^127^ A gold nanoparticles (AuNPs) carrying anti-EBV-miR-BART7-3p can silence the expression of miR-BART7-3p, thus reducing tumor growth in animal models.^128^ Besides, a monoclonal antibody targeting BARF1 also showed therapeutic activity in preclinical models of EBV^+^ NPC.^129^ Multiple small molecules, such as gemcitabine, sodium butyrate, and histone deacetylase inhibitors, are also capable of reactivating EBV lytic cycle via inducing the expression of BZLF1. Combining gemcitabine and valproic acid (GCb + VPA) can reactivate latent EBV into its lytic phase and upregulate the expression of BGLF4 and BXLF4 in tumor cells, which then convert anti-viral prodrug ganciclovir (GCV) into its active form, killing EBV^+^ tumor cells and inhibiting the replication of EBV. This combination therapy is also known as cytolytic virus activation therapy (CLVA).^130^ A phase I/II clinical trial has demonstrated the efficacy and safety profile of CLVA in real world NPC cohort.^102^

Activating anti-EBV immune response is another effective way of eliminating EBV positive NPC. C2@mLMP2 is a LMP2-mRNA lipid nanoparticle that can be delivered to tumor-draining lymph nodes and exhibited transfection efficiency. This nanoparticle can reverse CD8^+^ T cell exhaustion and exhibit synergistic anti-tumor effect with anti-PD1 therapy in animal models.^131^ Besides, a recombinant vaccinia virus encoding an EBNA1/LMP2 fusion protein called MVA-EL can also enhance T cell immunity in a dose dependent manner.^101^

## Discussion

NPC, a human malignancy with marked regional endemicity, has underwent significant advances in treatment strategies, leading to substantially improved patient long-term survival and quality of life. In this review, we systematically summarized recent advances in resolving the above-mentioned problems via targeting EBV, with the aim of providing a theoretical foundation for future research, proposing potential directions and highlighting remaining issues.

Plasma EBV DNA, a minimally invasive and easily accessible clinical liquid biopsy indicator, strongly predicts the occurrence and long-term prognosis of patients with NPC. The predictive power of circulating plasma EBV DNA is further augmented when combined with TNM staging system or image-based evaluation criteria, and serves to guide tailored adaptive treatments, which optimize treatment efficacy while reducing toxicity. However, optimum plasma EBV DNA cut-off value for the prognostic prediction and adaptive treatment of NPC is highly heterogeneous between different studies, which can be partially attributed to the inconsistency of operation protocol and detection methods of EBV DNA. Further studies should focus on unifying the standardized workflow of plasma EBV DNA detection of patients with NPC. In the meantime, multi-center large-scale clinical trials guided by a standardized workflow should also be conducted to mitigate the potential confounding effects and propose optimum cut-off value of plasma EBV DNA quantification in prognostic assessment and personalized treatment of NPC. Apart from plasma EBV DNA, serum EBV antibodies serve as another NPC screening biomarker. Combination of multiple serological EBV antibodies can achieve comparable prediction efficacy as plasma EBV DNA. However, the head-to-head comparisons between serum EBV antibody-based and plasma EBV DNA-based NPC screening models are still scarce and the health economic evaluation has not been formally conducted. Besides, the development of prognostic assessment and adaptive treatment systems based on serum EBV antibody profiles also remain inadequate. Furthermore, most of the clinical trials investigating the efficacy of EBV-related biomarkers for NPC screening or prognostic assessment were conducted in endemic regions, with only limited attention on non-endemic areas. The comparison of the utility of these EBV-related biomarkers across different ethnicities or populations with varying risks of NPC remains unexplored. In summary, plasma EBV DNA serves as a cornerstone for screening, prognostic prediction, and adaptive treatment guidance of NPC, yet requires standardized detection framework and validated, consistent cut-off value. Serum EBV antibodies offer complementary efficacy, but their clinical utility needs further development.

Despite significant therapeutic advances in locoregionally advanced patients with NPC, some of them remain unresponsive. EBV-targeted treatment strategies serve as a promising alternative for these patients. However, the therapeutic efficacy of EBV-CTLs infusion has been inconsistent across clinical trials, development and clinical validation of EBV-targeted CAR-T and TCR-T cell therapy products are mostly ongoing, and the discovery of small-molecule therapies remains at preliminary stage. Therefore, further preclinical and clinical evidence is required to support the clinical application of these strategies.

It is worth noticing that EBV vaccine is another promising yet immature field of developing NPC prevention and treatment therapies. EBV-protein based vaccine contains the essential viral components necessary for eliciting both humoral and cytotoxic immune response and numerous studies have illustrated their efficacy of blocking EBV infection and preventing the occurrence of NPC in preclinical models.^107^^,^^132^, ^133^, ^134^ In contrast, only limited studies have explored the potential of EBV mRNA vaccines in NPC prevention.^135^^,^^136^ Despite the lack of clinical evidence and relatively early stage of investigation, the future of developing more precise and efficacious EBV vaccines holds substantial clinical value.

In conclusion, EBV and its associated molecules serve as promising targets in a spectrum of clinical scenarios of NPC, from screening, prognostic assessment, and adaptive treatments to the development of cell therapies and other novel treatment strategies. Continued research to fully elucidate the roles of EBV in the oncogenesis of NPC and to translate these findings into clinical practice will be the key to fully realize their therapeutic potential.

## Contributors

MJ and LN contributed to the conceptualization and project administration of this review. FSY and SRJ contributed to the investigation, data curation, visualization, and writing original draft of this review. MJ and LN were involved in the supervision, review, and editing of the manuscript. All authors approved the final draft and accepted responsibility to submit for publication.

## Declaration of interests

There are no conflicts of interest associated with this paper.
